# Revolutionizing Underwater Sensor Performance: Tackling Rayleigh Scattering Challenges by Pseudo Random Noise

**DOI:** 10.1002/advs.202411967

**Published:** 2024-12-04

**Authors:** Qihao Hu, Fan Shang, Lina Ma, Wujie Wang, Yi Yu, Yujie Bian, Xiaoqian Zhu, Junqiang Song

**Affiliations:** ^1^ College of Meteorology and Oceanography National University of Defense Technology Changsha Hunan 410073 China

**Keywords:** fiber optic interferometric sensors, phase modulation, pseudorandom noise codes, Rayleigh scattering

## Abstract

Traditionally, Rayleigh scattering is thought to only impact fiber sensing system performance when the leading fiber is over 10 km long. However, this report illustrates theoretically and experimentally that Rayleigh scattering cannot be ignored in fiber optic interferometric sensor (FOIS) even with several hundred‐meter common leading fiber because of the interaction of Rayleigh backward scattering (RBS) and returning interference signal. Herein, a conceptual framework is developed to elucidate the interaction between RBS and FOIS interference, revealing that, beyond laser monochromacity, the self‐correction characteristic of laser pulses also influences coherent superposition. Building upon this novel insight, a phase modulation method based on pseudorandom noise (PRN) code is first proposed to address coherent RBS stacking on returning FOIS interferences while preserving high laser monochromacity. By modulating the interrogation pulses, a 21.3 dB suppression of background phase noise is achieved in FOIS with 3.3 km leading fiber. This study offers a holistic understanding of Rayleigh scattering in the leading fiber, encompassing experimental observations, theoretical modeling, physics analysis, and its resolution, thereby contributing to advancements in underwater sensing to broaden the understanding of the underwater environment.

## Graphical abstract

This paper is an innovative combination of spread spectrum communication technology and optical fiber sensing technology. The interdisciplinary study provides a promising strategy to address the Rayleigh scattering‐induced parasitic in large‐scale fiber sensing system.

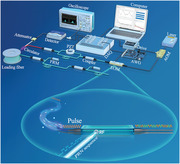

## 1 Introduction

The fiber optic interferometric sensors (FOISs) have emerged as a pivotal tool for exploring the undersea environment,^[^ ^1^, ^2^, ^3^, ^4^, ^5^ ^]^ offering significant advantages such as high sensitivity, seamless multiplexing for large‐scale arrays, natural remote transmission, and immunity to electromagnetic disturbances. Within this domain, the fiber optic hydrophone^[^ ^6^, ^7^, ^8^, ^9^, ^10^, ^11^, ^12^, ^13^ ^]^ is recognized as a promising solution for towed and sea‐bottomed arrays, while the fiber grating ocean bottom seismometer^[^ ^14^, ^15^, ^16^, ^17^, ^18^, ^19^ ^]^ stands as the cornerstone of the largest permanent reservoir monitoring network. Additionally, distributed acoustic sensing (DAS)^[^ ^1^, ^3^, ^20^, ^21^, ^22^, ^23^ ^]^ facilitates the capture of faint seismic signals from distant seabeds.

To optimize underwater reliability,^[^ ^12^ ^]^ minimize dimensions,^[^ ^24^ ^]^ achieve larger multiplexing arrays,^[^ ^25^, ^26^, ^27^ ^]^ and enhance sensing resolution,^[^ ^28^ ^]^ path‐matched differential interference^[^ ^29^ ^]^ structures are typically favored. These structures, exemplified by reflected‐optical fiber Michelson interferometer arrays^[^ ^8^, ^25^ ^]^ and fiber grating hydrophone arrays,^[^ ^15^, ^27^, ^30^, ^31^ ^]^ substantially reduce the number of underwater fiber components. Ordinarily, the interrogation module housing the laser and other optical and electrical components is connected to the underwater sensing array via a leading fiber. A simplified underwater configuration can be achieved when light travels up and down the same leading fiber, representing a promising setup for next‐generation applications. This configuration, termed common leading fiber, contrasts with non‐common leading fiber setups, where light propagates up and down in different leading fibers. Examples of common leading fiber structures include the aforementioned fiber grating hydrophone arrays^[^ ^15^, ^30^, ^31^ ^]^ and DAS.^[^ ^1^, ^3^ ^]^ However, while the simplified undersea structure offers numerous advantages, it also introduces several technical challenges, notably the persistent issue of Rayleigh scattering (RS).

RS is a pervasive phenomenon in fibers due to microscopic irregularities.^[^ ^32^, ^33^, ^34^ ^]^ The parasitic interferences between Rayleigh backscattering (RBS) and backward propagating interference signals occur when RBS light in the leading fiber coincides with sensor interferences, which could lead to various issues including heightened background phase noise,^[^ ^35^ ^]^ false channel crosstalk (i.e., the introduction of RS induce detected signal from target channel to others),^[^ ^36^ ^]^ and demodulation instability.^[^ ^37^ ^]^ Traditionally, it has been assumed that RS significantly impairs sensing performance only when the leading fiber exceeds 10 km.^[^ ^35^ ^]^ Consequently, research efforts have predominantly concentrated on remote sensing systems.^[^ ^25^, ^35^, ^38^, ^39^, ^40^, ^41^, ^42^ ^]^ The laser phase and frequency modulation approach has garnered attention for its ability to achieve noise suppression of up to 20 dB in a 25‐km leading fiber.^[^ ^35^ ^]^ Similarly, the phase‐generated‐carrier (PGC) modulation method, aimed at mitigating random phase drift,^[^ ^40^ ^]^ can suppress noise by up to 11.7 dB in a 400‐km leading fiber.^[^ ^38^ ^]^ Underwater path‐matched compensation interferometers offer relatively low background phase noise in a 50‐km leading fiber compared to dry‐end scenarios.^[^ ^39^ ^]^ These studies primarily focus on FOIS with non‐common leading fibers, utilizing the double Rayleigh scattering (DRS) model.^[^ ^35^, ^38^ ^]^ Because DRS is far weaker than RS, its impact on systems with short leading fibers is typically disregarded.

**Figure 1:**
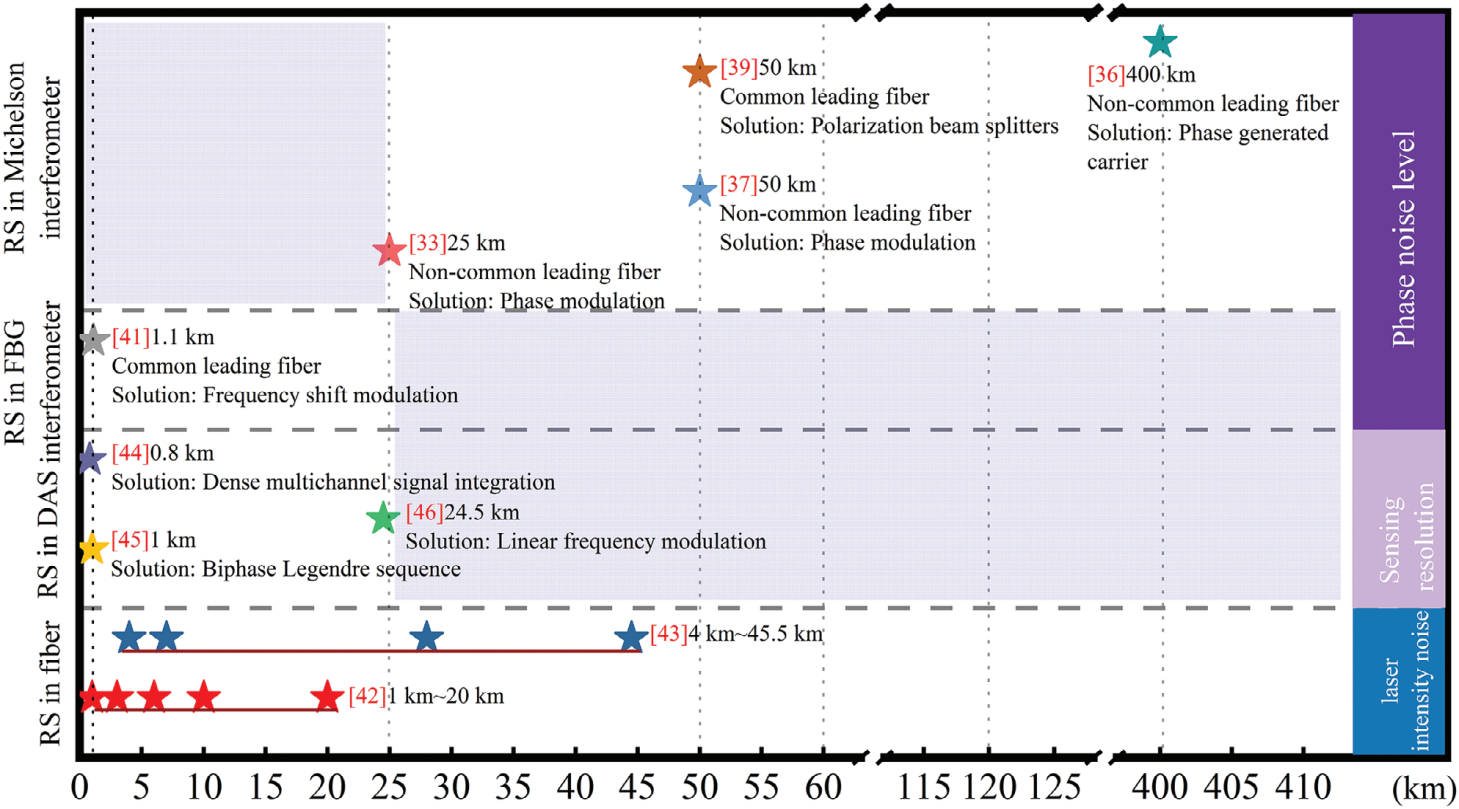
A brief summary of RS in single fibers, DAS, FBGs, and Michelson interferometers.

However, in FOIS arrays with common leading fibers, the collision of RBS with the up‐leading interference can degrade system performance.^[^ ^36^, ^37^ ^]^ By incorporating polarization beam splite into a Michelson interferometer equipped with a 50‐km sensing fiber, an enhancement of the signal‐to‐noise ratio by 6.69 dB has been observed.^[^ ^41^ ^]^ Studies show that there is a sublinear growth in RS‐induced intensity noise in km level leading fiber when laser noise is taken into consideration.^[^ ^44^, ^45^ ^]^ Even more, the robust interferometric fiber Bragg gratings (FBGs) sensing arrays cannot be applied even with 1.1 km leading fiber if frequency modulations are absent.^[^ ^43^ ^]^ Furthermore, Coherent RBS emerges as a primary factor limiting spatial resolution in DAS, with dense multichannel signal interrogation^[^ ^46^ ^]^ and biphase Legendre sequence^[^ ^47^ ^]^ methods employed to enhance resolution within 1 km. **Figure** **1** provides an overview of RS in single fibers, DAS, FBGs, and Michelson interferometers.

Figure 1 highlights two significant gaps that require attention. One pertains to the utilization of RS in fiber Michelson interferometers with a leading fiber shorter than 10 km, while the other concerns RS in FBG interferometers (i.e., The sensing channels consist of FBGs) and DAS systems with a transmission distance exceeding 50 km, representing a technical bottleneck between long‐range capability and high resolution. Our primary focus lies on addressing the former gap, necessitating a more precise understanding of the interaction between RS and effective interference.

This study comprehensively examined the often‐neglected phenomenon of RBS in FOIS employing short common leading fibers. A parasitic interference model elucidating the interplay between RBS and effective interference in FOIS was introduced, which distinguishes itself from the established DRS model.^[^ ^35^, ^38^ ^]^ Given the higher intensity of RBS compared to DRS, the impact of RBS was asserted to be more significant even with a brief leading fiber. Observations revealed a 15.7 dB increase in background noise attributed to the leading fiber when it spanned 300 meters, and a maximum measured noise elevation of 33.6 dB observed with a 3.3 km leading fiber.

The simple and reliable structure exhibits significant potential however it simultaneously introduces substantial phase noise induced by RBS. Unfortunately, the aforementioned methods are not applicable for addressing the issue in FOIS with common leading fiber due to the absence of a solution that reconciles high laser monochromacy and short coherence length. Here, strategies for mitigating RBS induced parasitic interference were explored. Beyond conventional laser monochromacy, the self‐correction feature of laser pulses was discovered to influence coherent superposition. Building on this insight, a pseudorandom noise (PRN) phase modulation technique based on spread‐spectrum communication was first proposed to manage the coherent superposition of RBS and effective FOIS interference while upholding high laser monochromacy. This approach achieved a noise suppression of 21.3 dB with a 3.3 km leading fiber.

As underwater applications increasingly pursue simple and reliable structures, utilizing a single fiber for both sensor array and transmission pathway emerges as the ultimate objective.^[^ ^48^ ^]^ The findings of this study highlight that RBS in such interferometric sensing systems cannot be disregarded even with a leading fiber shorter than 1 km. To overcome this impasse, an ingenious solution named PRN phase modulation to this problem has been proposed theoretically and then demonstrated experimentally. The reported case of RS suppression in FOIS with a common leading fiber is the first to be documented based on physical mechanisms. The method is probably the optimal scheme due to the little negative impact. These findings not only offer a novel avenue for further advancements in interferometric system but also have implication for the development of DAS.

## 2 Results and Discussion

### 2.1 Non‐Negligible RBS in FOIS with Short Common Leading Fiber: Its Physical Origin and a Relative Short Length

**Figure 2:**
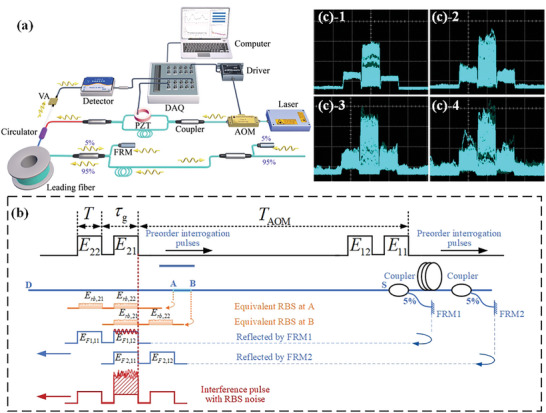
FOIS Schematic diagrams. a) Schematic diagram of sensing system. The output voltage waveforms can be captured and displayed by the oscilloscope. b) Superposing procedure of returned FOIS interference and RBS. *T*_AOM_is adjustable within the range from 20 to 250 kHz. The PGC modulation frequency is 1/16 of AOM output frequency, τ_*g*_= 500 ns, and *T* is adjustable from 100 to 490 ns. c) The oscilloscope waveforms of the pulse sequences with 0, 2, 4, and 8 RBS‐CSs. The middle pillar on the oscilloscope screen, characterized by a more pronounced rising‐edge jitter compared to others, signifies the magnitude of interference.

**Figure** **2**a depicts the system structure of opto‐electro devices in FOIS. A narrow‐linewidth continuous‐wave laser is utilized to minimize frequency noise. The continuous laser is modulated into pulses with a width of *T* and a repetition period of *T*_AOM_by an acoustic‐optic modulator (AOM).

The outgoing laser pule *E*_1_ could be divided into to two pulses *E*_11_and*E*_12_with a time interval of τ_*g*_ when it passes through a fiber Mach‐Zander interferometer (MZI) with the optical path difference Δ*l*_M_ between two arms. A portion of the fiber in the short arm is wound around a piezoelectric (PZT) ring to generate the high‐frequency PGC signal along with the pre‐pulse *E*_11_. Subsequently, this pulse pair is transmitted into the fiber Michelson interferometer‐based sensor through a circulator and a long leading fiber. The sensor, comprising two 5:95 fiber couplers and two fiber Faraday rotator mirrors (FRMs) are constructed to eliminate polarization induced signal fading.^[^ ^49^ ^]^ After passing through the first coupler, both *E*_11_and *E*_12_could be split into two pulses and there will be four sub‐pulses reflected by FRM1 (i.e., *E*_*F*1,11_, *E*_*F*1,12_) and FRM2 (i.e., *E*_*F*2,11_and *E*_*F*2,12_).

Given that the optical path difference between two FRMs is $\Delta l_{M}/2$, the round‐trip time delay of the two FRMs, which corresponds to the MZI, is τ_*g*_. Thereby, the path difference of the interrogation pulse pair is matched, facilitating effective interference. The received pulses are attenuated by a variable attenuator and subsequently converted into electrical signals via the detector. A data acquisition (DAQ, USB‐6361) is employed to sample the interferences, providing the synchronization driving signals for the AOM and the PZT. Finally, the sampled interference signals are demodulated and analyzed by computer.

Figure 2b illustrates how the interferences were impacted by RBS. As the time delay between the two FRMs matches the interval of the interrogation pulse pair, the second pulse reflected by FRM1 (denoted as *E*_*F*1,12_) overlaps with the pre‐pulse reflected by FRM2 (denoted as *E*_*F*2,11_). The combination of the two pulses gives rise to the primary interference of the FOIS, carrying the desired sensing signal. Simultaneously, the latter outcoming pulse pair from MZI (*E*_21_and*E*_22_) is launched into the leading fiber, inducing RBS along the behind leading fiber.

The point A and B represent the intersection points of interference and pulse pair (*E*_21_and*E*_22_), respectively. The RBS of*E*_21_ (denoted as *E*_*rb*,21_) overlaps with *E*_*F*1,12_ and *E*_*F*2,11_ at point B and the RBS of *E*_22_(denoted as *E*_*rb*,22_) overlaps with *E*_*rb*,21_, *E*_*F*1,12_, and*E*_*F*2,11_at point A. Superposition of the four pulses initiates at the encounter point of two oppositely propagating pulse pairs and terminates when they completely bypass each other. Despite RBS occurring along the entire fiber, it only affects the response of FOIS when the upward‐propagating FOIS interference encounters the downward‐propagating interrogation pulses because voltage values were collected only once for each backward‐propagating interference signals. This contact zone is termed the effective RBS collision segment (RBS‐CS).

Not accounting for transmission loss, the injected two laser pulse pairs at point D can be expressed as follows:

(1) $$\left\{\begin{array}{c} E_{11}\left(t\right)=E_{0}\cdot e^{j\left[\omega t+\varphi_{n}\left(t\right)+C\cos(2\pi f_{\mathrm{PGC}}t)\right]}\\ E_{12}\left(t\right)=E_{0}\cdot e^{j\left[\omega t+\varphi_{n}\left(t\right)+\omega \tau_{g}\right]}\\ E_{21}\left(t\right)=E_{0}\cdot e^{j\left[\omega t+\varphi_{n}\left(t\right)+C\cos(2\pi f_{\mathrm{PGC}}t)+\omega T_{\mathrm{AOM}}\right]}\\ E_{22}\left(t\right)=E_{0}\cdot e^{j\left[\omega t+\varphi_{n}\left(t\right)+\omega \tau_{g}+\omega T_{\mathrm{AOM}}\right]} \end{array}\right.$$

where *E*_0_ denotes the electric field amplitude of the light wave, ωdenotes the light wave frequency, *f*_PGC_ denotes the PGC modulation frequency, φ_*n*_(*t*) denotes the random phase noise of source and *C* denotes the amplitude of PGC demodulation.

The backward‐propagating pulses *E*_*F*2,11_,*E*_*F*1,12_,*E*_*rb*,21_, and *E*_*rb*,22_at point A can be expressed as follows:

(2) $$\left\{\begin{array}{c} E_{F1,12}\left(t\right)=r_{1}E_{12}\left(t\right)e^{j\varphi_{12}}\\ E_{F2,11}\left(t\right)=r_{2}E_{11}\left(t\right)e^{j\left[\varphi_{s}\left(t\right)+\varphi_{11}\right]}\\ E_{rb,21}\left(t\right)=R_{rb}E_{21}\left(t\right)e^{j\varphi_{21}}\\ E_{rb,22}\left(t\right)=R_{rb}E_{22}\left(t\right)e^{j\varphi_{22}} \end{array}\right.$$

where φ_11_, φ_12_, φ_21_and φ_22_ represent the propagating phase delay of the leading fiber from D through S to FRM2 and back to A, D through S to FRM1 and back to A, D to B and back to A and D to A, respectively. φ_*s*_(*t*) and *R_rb_* represent the perturbation phase at the sensing channel (the fiber between two FRMs) and the equivalent RBS coefficient, respectively. *r*_1_ and *r*_2_ denote the equivalent reflectivity demonstrating the light amplitude loss returned from FRM1 and FRM2, respectively. The interference generated by these mixed pulses at point A can be expressed as follows:

(3) $$\begin{array}{cc} I\left(t\right) & =〈\left[E_{F1,12}\left(t\right)+E_{F2,11}\left(t\right)+E_{rb,21}\left(t\right)+E_{rb,22}\left(t\right)\right]\\ & \;\times {\left[E_{F1,12}\left(t\right)+E_{F2,11}\left(t\right)+E_{rb,21}\left(t\right)+E_{rb,22}\left(t\right)\right]}^{*}〉\\ & =\langle E_{F1,12}\left(t\right)E_{F1,12}^{*}\left(t\right)\rangle +\langle E_{F2,11}\left(t\right)E_{F2,11}^{*}\left(t\right)\rangle \\ & \;+\langle E_{rb,21}\left(t\right)E_{rb,21}^{*}\left(t\right)\rangle +\langle E_{rb,22}\left(t\right)E_{rb,22}^{*}\left(t\right)\rangle \\ & \;+\langle 2Re\left[E_{F1,12}\left(t\right)E_{F2,11}^{*}\left(t\right)\right]\rangle +\langle 2Re\left[E_{F1,12}\left(t\right)E_{rb,21}^{*}\left(t\right)\right]\rangle \\ & \;+\langle 2Re\left[E_{F1,12}\left(t\right)E_{rb,22}^{*}\left(t\right)\right]\rangle +\langle 2Re\left[E_{F2,11}\left(t\right)E_{rb,21}^{*}\left(t\right)\right]\rangle \\ & \;+\langle 2Re\left[E_{F2,11}\left(t\right)E_{rb,22}^{*}\left(t\right)\right]\rangle +\langle 2Re\left[E_{rb,21}\left(t\right)E_{rb,22}^{*}\left(t\right)\right]\rangle \end{array}$$

where〈〉represents the time‐series sliding mean operation, primarily determined by the frequency‐band response of the photodetector. Interference terms lacking PGC carriers are filtered out by low‐pass filtering during PGC demodulation,^[^ ^40^ ^]^ and the intensities of the two RBS terms are much lower than those reflected by FRMs. Consequently, the equation above can be further simplified as follows:

(4) $$\begin{array}{c} \begin{array}{cc} I\left(t\right) & =I_{DC}\left(t\right)+\langle 2Re\left[E_{F1,12}\left(t\right)E_{F2,11}^{*}\left(t\right)\right]\rangle \\ & \;+\langle 2Re\left[E_{F1,12}\left(t\right)E_{rb,21}^{*}\left(t\right)\right]\rangle +\langle 2Re\left[E_{F2,11}\left(t\right)E_{rb,22}^{*}\left(t\right)\right]\rangle \\ & =I_{DC}\left(t\right)+2r_{1}r_{2}\cos\left[C\cos\left(2\pi f_{\mathrm{PGC}}t\right)+\varphi_{s}\left(t\right)\right]\\ & \;+2r_{1}\langle Re\left(R_{rb}\right)\rangle \cos\left[C\cos\left(2\pi f_{\mathrm{PGC}}t\right)+\varphi_{12}-\varphi_{21}\right]\\ & \;+2r_{2}\langle Re\left(R_{rb}\right)\rangle \cos\left[C\cos\left(2\pi f_{\mathrm{PGC}}t\right)+\varphi_{s}\left(t\right)+\varphi_{11}-\varphi_{22}\right] \end{array} \end{array}$$

The second term in the equation above denotes the expected FOIS response. Because of RBS, two parasitic interference results are superimposed on the original interference, leading to the following two consequences. First, the non‐linearity of the PGC demodulation algorithm causes demodulation instability,^[^ ^31^ ^]^ directly increasing background phase noise. Second, disturbances transmitted on the leading fiber between A and S introduce additional noise into the demodulated result, further elevating the phase noise level.

The theory above illustrates the scenario when one effective RBS‐CS is considered in the leading fiber. With an increase in the length of the leading fiber, more effective RBS‐CSs emerge at intervals of $d=c/\left(2n\right)\cdot T_{\mathrm{AOM}}$, resulting in additional RBS‐induced parasitic interferences superimposed on the FOIS response. For a leading fiber with a length of *L*, the number of effective RBS‐CSs can be calculated using the following expression:

(5) $$N=\left\lfloor L/\left(\frac{c}{2n}T_{\mathrm{AOM}}\right)\right\rfloor$$

where ⌊⌋ is the floor operator.

Figure 2c depicts the recorded images of the returned laser pulses with different numbers of effective RBS‐CSs in the leading fiber. The leading fiber utilized here is a 3.3‐km bending‐insensitive single‐mode fiber of BI15‐80‐U16, with the interrogation frequency set to 20, 40, 140, and 250 kHz, corresponding to effective RBS‐CS numbers of 0, 1, 4, and 8, respectively. The original non‐interfered pulse gradually experiences intensity fluctuations as the number of effective RBS‐CSs increases, while significant fluctuations are observed within the width range of the original interference pulse.

Because the PGC modulation is typically set at 1/16 of the interrogation frequency, changes in the interrogation frequency can also affect the demodulated background phase noise. Generally, higher interrogation frequencies result in lower background phase noise levels. We maintain a fixed interrogation frequency of 125 kHz and vary the length of the leading fiber to 2, 300, 500, 1300, 1700, and 3300 m, respectively, resulting in corresponding effective RBS‐CS numbers of 0, 0, 1, 4, 5, and 8.

**Figure 3:**
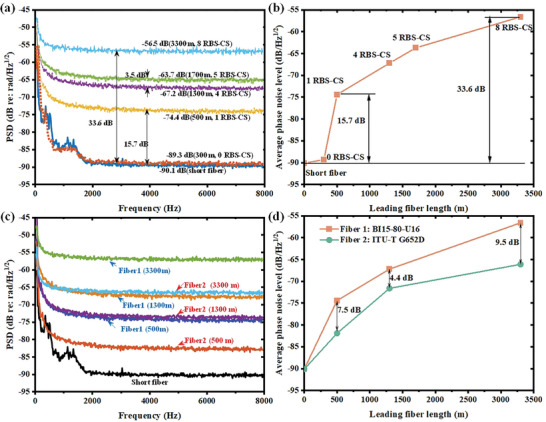
The demodulation results of sensing system. a) Phase noise frequency spectrum using BI15‐80‐U16. The number of RBS‐CSs is adjusted by pulse frequency and fiber length. b) Phase noise at 4 kHz using BI15‐80‐U16. c) Comparison of phase noise frequency spectrum using BI15‐80‐U16 and ITU‐T G652D. d) Comparison of phase noise at 4 kHz using BI15‐80‐U16 and ITU‐T G652D.

The averaged background phase noise level over 240 demodulations is depicted in **Figure** **3**a, clearly demonstrating a positive correlation between the number of effective RBS‐CSs and the demodulated background phase noise level. When the leading fiber measures 2 or 300 m in length, lacking effective RBS‐CSs, the demodulation background phase noise hovers around −90 $\mathrm{dB}/\sqrt{\mathrm{Hz}}@$4 kHz. The appearance of the first effective RBS‐CS raises the background phase noise by ≈15.7 dB. With 4, 5, and 8 effective RBS‐CSs, the corresponding background phase noise increases by about 22.9, 26.5, and 33.6 dB, respectively, as depicted in Figure 3b.

Figure 3c compares cases using bending‐insensitive and sensitive fibers as the leading fiber. Utilizing a bending‐sensitive fiber ITU‐T G652D as the leading fiber while varying its length from 500, 1400, to 3300 m, the background phase noise levels at 4 kHz are −81.9 $\mathrm{dB}/\sqrt{\mathrm{Hz}}$, −72.8 $\mathrm{dB}/\sqrt{\mathrm{Hz}}$, and −66.0 $\mathrm{dB}/\sqrt{\mathrm{Hz}}$, respectively, corresponding to 1, 4, and 8 effective RBS‐CSs. Compared to the shortest fiber scenario, the noise levels induced by RBS are 8.2 dB at 500 m, 17.3 dB at 1400 m, and 24.1 dB at 3.3 km. Figure 3d indicates a difference of 4.4–9.5 dB between the two types of leading fibers, with the bending‐sensitive fiber inducing less RBS noise. The differences arise from the isotropic nature of RS, whereby RBS, as a component of RS, can be captured and transmitted in a backward direction along the fiber axis determined by the numerical aperture (NA) of the fiber. A larger NA results in stronger RBS, and generally, the bending‐insensitive fiber has a larger NA than the non‐bending fiber, thus exhibiting stronger RBS. This further validates the parasitic interference theory discussed earlier.

Both theoretical and experimental results demonstrate that even with a leading fiber shorter than 1 km, RBS can significantly degrade FOIS performance. The distinction between a long and short leading fiber is relative. For instance, a 3.3 km leading fiber may be considered short when the interrogation frequency is 20 kHz. However, when the interrogation frequency reaches 250 kHz, 3.3 km becomes a considerable distance. The parasitic interference between the upward‐propagating returned FOIS response and RBS elucidates why the system noise level increases due to RBS.

Because RS cannot be fundamentally eliminated, the solution lies in controlling parasitic interference. Two potential solutions can be considered. One is using a low interrogation frequency, which ultimately limits the signal frequency of interest, such as the FOIS sensing frequency, making it unsuitable for underwater applications with increasingly longer fibers. Another approach involves reducing the interrogation laser coherence length to avoid coherent superposition. However, a lower laser coherence length results in a wider laser linewidth and higher laser frequency noise, potentially deteriorating the system background noise level.^[^ ^50^, ^51^, ^52^ ^]^ The only solution left is *R_rb_*.

### 2.2 *R_rb_*: A Key Identification of Parasitic Interference Related to the Laser Self‐correlation Function

**Figure 4:**
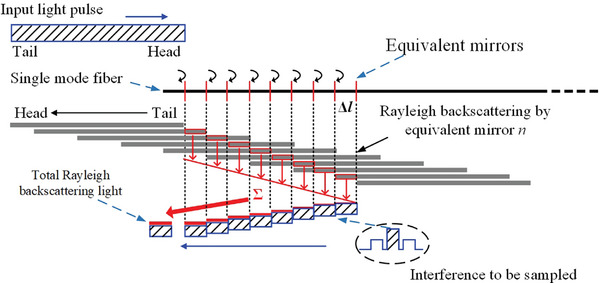
Model of the superposition procedure of FOIS response and RBS. The dark gray rectangle array represents the RBS in leading fiber at consecutive time points. The overlapped RBS and up‐propagating interference pulses are represented by red and blue shading bar, respectively.

In the above parasitic interference model, the simplification of RBS and FOIS response superposition as three light waves is a practical simplification. However, in practice, this process is more intricate. For one sampling point within the coherent pulse, the theoretical model depicted in **Figure** **4** applies to the entire returned interference pulses, including the non‐interfered pulses before and after the interference pulse.

In the fiber, RS points are continuous and can be likened to a series of reflection plates. Assuming the acquisition time for one sample point is Δ*t*, and for an analog to digital converter with a sampling rate of 100 MHz, Δ*t* translates to several to several tens of nanoseconds. When the up‐propagating interference pulse encounters the down‐propagating interrogation pulse pair, the RBS stimulated by the latter overlays the former. As they travel in opposite directions, the contact time of the up‐ and down‐propagating pulses is *T* /2 where *T* represents the width of an individual interrogation pulse, and Δ*t* can be disregarded because it is considerably smaller than *T*. Superposition occurs in the fiber over a length of *Tv_g_*/2, where *v_g_* denotes the group velocity of light in the fiber. This length of fiber where superposition occurs is equivalent to *N* mirror plates with spacing Δ*l* = Δ*tv_g_*/2. Assuming the reflectivity of each equivalent mirror is *r_rb_*, and the additional reflected phase is ϕ_*rb*_. Neglecting the slight transmission loss in the fiber, the total RBS electric field component *E*_*rb*,2*i*_(*t*) can be expressed as:

(6) $$\begin{array}{ccc} E_{rb,2i}\left(t\right) & = & h_{0}E_{2i}\left(t\right)+h_{1}E_{2i}\left(t-\Delta t\right)+h_{2}E_{2i}\left(t-2\Delta t\right)+\cdots \\ & = & r_{rb}e^{\left(-i\phi_{rb}\right)}\sum_{n=0}^{N-1}\;E_{2i}\left(t-n\cdot \Delta t\right).\;\left(i=1,2\right) \end{array}$$

In Equation (6), the impact responses of all equivalent Rayleigh reflector lenses are assumed to be identical, i.e., $h_{0}=h_{1}=\cdots =h_{N-1}=r_{rb}e^{\left(-i\phi_{rb}\right)}$. The third parasitic interference term in Equation (4) can be expanded as:

(7) $$\begin{array}{cc} & \langle 2Re\left[E_{F2,11}\left(t\right)E_{rb,22}^{*}\left(t\right)\right]\rangle =2r_{2}\cos[C\cos\left(2\pi f_{\mathrm{PGC}}t\right)+\varphi_{11}-\varphi_{22}\\ & \;+\varphi_{s}\left(t\right)-\varphi_{0}]Re\left(r_{rb}\sum_{n=0}^{N-1}\langle E_{11}\left(t\right)E_{11}^{*}\left(t-n\cdot \Delta t\right)\rangle \right) \end{array}$$

whereφ_0_ = ωτ_*g*_ + ω*T*_AOM_ − ϕ_*rb*_, representing the working point of this interference term. The notation *R_e_* (τ) denotes the autocorrelation function of the light pulse sequence:

(8) $$R_{e}\left(\tau \right)=\langle E\left(t\right)\cdot E^{*}\left(t-\tau \right)\rangle =\frac{1}{T}\int_{-T/2}^{T/2}E\left(t\right)\cdot E^{*}\left(t-\tau \right)dt.$$

Equation (7) can be further expressed as follows:

(9) $$\begin{array}{cc} & \langle 2Re\left[E_{F2,11}\left(t\right)E_{rb,22}^{*}\left(t\right)\right]\rangle =2r_{2}\cos[C\cos\left(2\pi f_{\mathrm{PGC}}t\right)+\varphi_{11}-\varphi_{22}\\ & \;+\varphi_{s}\left(t\right)-\varphi_{0}]Re\left[Nr_{rb}\sum_{n=0}^{N-1}R_{e}\left(n\cdot \Delta t\right)\right]\\ & \;=2r_{2}R_{rb}\cos\left[C\cos\left(2\pi f_{\mathrm{PGC}}t\right)+\varphi_{11}-\varphi_{12}+\varphi_{s}\left(t\right)-\varphi_{0}\right], \end{array}$$

where $R_{rb}=Nr_{rb}Re\left[\sum_{n=0}^{N-1}R_{e}\left(n\cdot \Delta t\right)\right]$, indicating that the total backward RS intensity is determined by the accumulation of RBS in a fiber of length *Tv_g_*/2 and the autocorrelation function of the interrogation light pulse.

Similarly, the second parasitic interference term in Equation (5) can be expressed as follows:

(10)

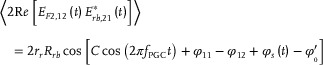

where 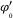 represents the working point of this interference term similar to φ_0_. Equations (9) and (10) demonstrate that the intensity of the parasitic interferences can be reduced by altering the autocorrelation properties of the light pulse *R_e_* (τ), offering a novel approach to suppress RBS. A method is established for reducing the autocorrelation properties of the laser without altering its monochromaticity, including the light wave phase. The interrogation light pulse with arbitrary phase modulation can be expressed as follows:

(11) $$E_{in}\left(t\right)=E_{0}\cdot e^{j\left[\omega t+\phi \left(t\right)+\varphi_{n}\left(t\right)\right]}$$

where ϕ(*t*) represents the phase modulation term, and ϕ(*t*) = 0 indicates no modulation.

**Figure 5:**
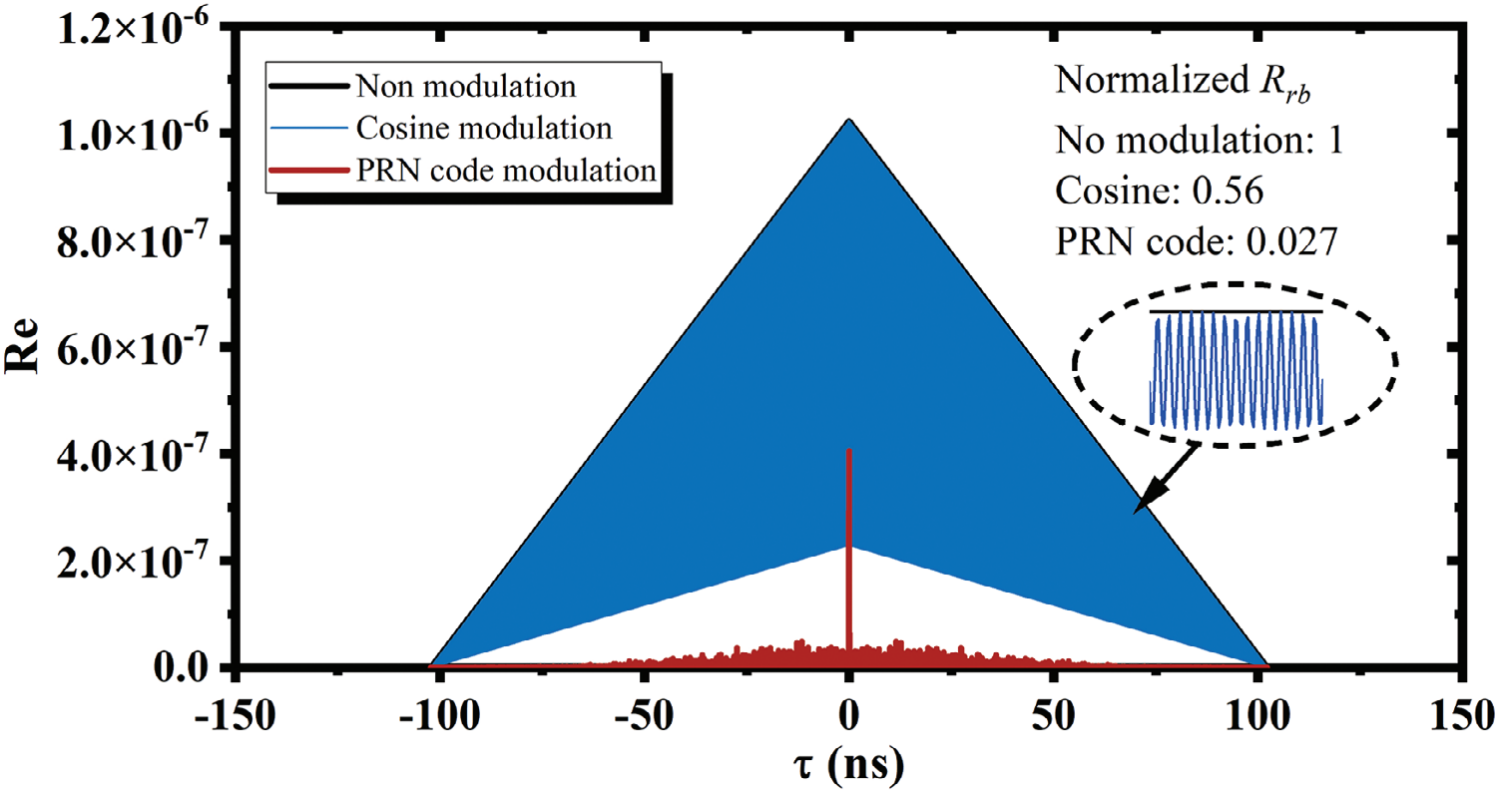
Simulated self‐correlation curves of the pulsed laser. The cosine modulation is ϕ(*t*) = cos (2π*f_m_t*) where *f_m_* = 1.63 MHz.

In order to highlight the superiority of PRN sequence in phase modulation compare the more intuitively, the self‐correlation curves of the pulsed laser with three modulation schemes are depicted in **Figure** **5** and the key simulation parameters are given in **Table** **1**.

**Table 1:** Simulation parameters.

Noise value	Perturbation	Wavelength	Pulse width	Sampling rate
π/5	1%	1550 nm	102.3 ns	10 MHz

The autocorrelation curve of the pulsed laser without any phase modulation is represented by the black solid line in Figure 5. The blue curve illustrates the autocorrelation curve of the pulsed laser with cosine modulation, where the modulation amplitude and frequency are 1 and 1.63 MHz, respectively. A cosine oscillation is evident in the autocorrelation function curve. The red curve depicts the autocorrelation curve of the pulsed laser with PRN modulation, and ϕ(*t*) can be expressed as follows:

(12) $$\phi \left(t\right)=\left\{\begin{array}{c} \pi \cdot \sum_{n=0}^{B}a_{\mathrm{PN}}\cdot p\left(t-nT_{c}\right),0<t<T\\ 0,\mathrm{otherwise} \end{array}\right.$$

where, *a*_PN_represents the PRN parameter with a value of {0, 1}. The chip interval $T_{c}=T/B$ is determined by the order of the pseudorandom sequence code. For a 7‐bit Gold code, *B* = 2^7^ − 1 = 127. *p*(*t*) is a rectangular pulse waveform function and can be expressed as:

(13) $$p\left(t\right)=\left\{\begin{array}{c} 1,0<t<T_{c}\\ 0,\mathrm{otherwise} \end{array}\right.$$

The red curve in Figure 5 illustrates a substantial decline in the autocorrelation of the laser pulse upon the application of PRN phase modulation, with the self‐coherence width narrowing to the order of nanoseconds. Given that the value of *R_rb_* is determined by the cumulative sum of all autocorrelation function values within the pulse width, various phase modulations can be normalized using the value *R_rb_* as a baseline, without any phase modulation. Consequently, a normalized value of 0.56 is obtained for cosine phase modulation, contrasting with 0.027 for PRN phase modulation. This discrepancy underscores the profound impact of phase modulation in mitigating the intensity of parasitic interference, yielding a maximum reduction of ≈4.7 dB for cosine modulation and an impressive 31 dB for PRN modulation. Moreover, while the demodulated phase noise level does not exhibit direct proportionality to interference intensity, the latter serves as a pivotal factor in dictating the suppression dynamics of the former.^[^ ^53^ ^]^ This simulation reveals the efficacy of both cosine and PRN modulation strategies, manifesting a maximum phase noise suppression effect of 4.7 dB for cosine modulation and a notable 31 dB for PRN modulation.

### 2.3 PRN Phase Modulation Method for RBS Suppression

**Figure 6:**
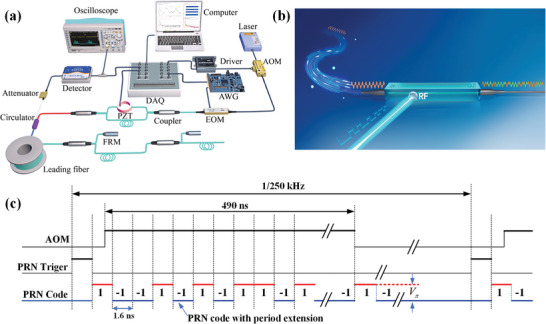
Schematic diagram of experimental system structure. a) FOIS with PRN mode modulation. b) Schematic diagram of light waves with PRN phase modulation. c) Timing structure of AOM and PRN modulation module, where *T*_AOM_= 4 µs, *T* = 490 ns.

Based on the system mentioned above, a PRN phase modulation module was integrated. An electro‐optic modulator (EOM) was positioned behind the AOM. The requisite PRN signal is generated by a signal generator, with the PRN sequence code crafted via computer software. Concurrently, the NIUSB‐6361 furnishes a synchronization trigger signal for PRN modulation. Experimental system configuration is depicted in **Figure** **6**a. Figure 6b illustrates the schematic diagram of a light wave with pseudorandom code phase modulation. Essentially, PRN phase modulation introduces a π shift on the continuous light wave according to the pseudorandom sequence code. Figure 6c displays the timing sequence of PRN modulation and AOM modulated signals. The laser operates with a pulse width of 490 ns and a repetition frequency of 250 kHz. Within each pulse width, the light‐wave phase changes either by zero or π phase at periodic intervals of *T_c_* = 1.6 ns.

**Figure 7:**
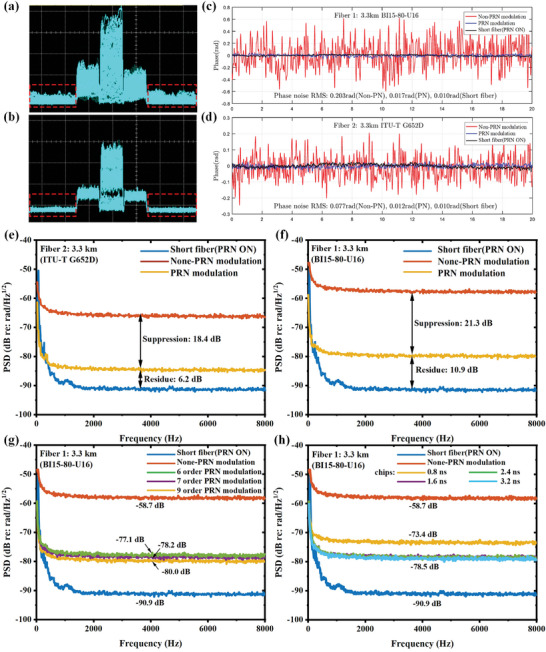
Comparison of demodulation results before and after PRN modulation. a,b) Images of interference without and with PRN modulation. c) Demodulated FOIS signals in the time domain using BI15‐80‐U16. d) Demodulated FOIS signals in the time domain using ITU‐T‐G652D. e,f) The background noise with and without PRN modulation under two types leading fiber. g) The background noises in BI15‐80‐U16 fiber with three types of Gold codes. h) The background noises in BI15‐80‐U16 with four chip intervals.

**Figure** **7**a,b showcases the oscilloscope images capturing the returned light pulses converted by the photoelectric detector after passing a 3.3‐km leading fiber of ITU‐T‐G652D. In the absence of FRM reflection pulses with PRN modulation inactive, the conspicuous light intensity fluctuations indicated the light generated by RBS. The first and third non‐interference pulses manifest as fluctuations due to the amalgamation of FRM‐reflected light and RBS. The primary interference pulse is notably intensified in intensity owing to coherent RBS superposition. PRN modulation leads to a substantial reduction in light intensity and fluctuations at each juncture, indicative of substantial suppression of parasitic interferences.

Figure 7a,b portrays the demodulated FOIS signals of BI15‐80‐U16 and ITU‐T‐G652D in the time domain, both with and without PRN modulation, utilizing a 3.3‐km leading fiber. Additionally, results obtained with a short leading fiber (≈2 m) are presented. Upon PRN modulation activation, the fluctuation range of demodulated noise amplitude (0.017 rad) aligns closely with that observed with the short leading fiber (0.01 rad), as depicted in Figure 7c. Conversely, in Figure 7d, these values are 0.012 and 0.01 rad, respectively. By contrast, with PRN modulation disabled in BI15‐80‐U16 and ITU‐T‐G652D, demodulated signal amplitudes increase to 0.203 and 0.077 rad, respectively.

Figure 7e–h depicts the average results in the frequency domain, with Figure 7e,f showcasing demodulated signals from a 3.3‐km leading fiber of BI15‐80‐U16 and ITU‐T‐G652D, respectively. As illustrated in Figure 7f, employing the 3.3‐km BI15‐80‐U16 leading fiber escalates the background phase noise level from −90.9 $\mathrm{dB}/\sqrt{\mathrm{Hz}}@$4 kHz to −58.7 $\mathrm{dB}/\sqrt{\mathrm{Hz}}@$4 kHz. The phenomenon is mitigated by employing PRN code modulation to achieve a suppression effect of ≈21.3 dB, with the signal attenuated to −80 dB. Analogously, when the 3.3‐km ITU‐T‐G652D fiber is utilized, the background phase noise of the hydrophone elevates from −90.9 dB@4 kHz to −66.3 $\mathrm{dB}/\sqrt{\mathrm{Hz}}@$4 kHz. With PRN code modulation, the background phase noise decreases to −84.7 $\mathrm{dB}/\sqrt{\mathrm{Hz}}@$4 kHz, resulting in a suppression of ≈18.4 dB.

PRN modulation employing the 6th, 7th, and 9th order Gold pseudorandom codes (with chip numbers of 63, 127, and 511, respectively) is conducted to probe the correlation between RBS noise suppression and pseudorandom sequence order. Throughout the experiment, the modulation chip interval remains constant at 1.6 ns, with each Gold code order necessitating periodic extension to slightly exceed the optical pulse width (490 ns). Utilizing the 3.3‐km BI15‐80‐U16 fiber as the leading fiber, the RBS noise suppression effects with the three Gold code variants are delineated in Figure 7g. The noise floor spectrum levels at 4 kHz for the 6th, 7th, and 9th order PRN sequences are −77.1, −78.2, and −80.0 dB, respectively. This observation underscores that, for a consistent chip interval, a higher‐order pseudorandom sequence with an extended fragment number delivers superior RBS suppression effects. This is attributed to the higher‐order Gold code sequences possessing mathematical statistical characteristics akin to Gaussian white noise, thereby featuring sharper correlation peaks in their autocorrelation function.

The PRN sequence code is maintained at the 7th order, while varying the chip interval to 0.8, 1.6, 2.4, and 3.2 ns respectively, respectively. The results, as depicted in Figure 7h, demonstrate slight differences in the measured background noise levels across the latter three chip intervals, averaging ≈−78.5 $\mathrm{dB}/\sqrt{\mathrm{Hz}}@$4 kHz, with an associated RBS suppression effect of 19.8 dB. Notably, the shortest 0.8 ns chip interval achieved slightly worse result, because the reduction of pulse coherence length influenced the main interference to some extent.

The experimental results support the reliability of the theoretical model. According to the theory mentioned above, the lower the correlation coefficients, the poorer the correlation among laser pulses, and the lower the background phase noise. Here, both increasing PRN fragment number and decreasing fragment length could further reduce the total correlation, which in turn leads to a decrease in phase noise, as shown in Figure 7g,h.

The occurrence of parasitic interference caused by RBS in FOIS utilizing a common leading fiber has been thoroughly examined. In contrast to situations involving distinct up and down leading fibers, the presence of RBS exert substantial influence within this configuration owing to optical interference effects, even when employing a relatively short leading fiber spanning just several hundred meters. The presence of RBS‐CS, which is directly proportional to the background phase noise, is collectively determined by the length of the leading fiber and the interrogation frequency.

In general, the next interrogation pulse must wait until all time‐divided multiplexing sensor responses have been received^[^ ^54^ ^]^ and the principle has also to be taken into account in FOIS arrays. But this principle inevitably constrains the sensing signal frequency of interest, attributable to the necessity of employing high laser monochromaticity for ultra‐low noise to fulfill the requirements of weak underwater acoustic signal detection, thus leading to an extended coherent length of the laser.

A physical depiction discussing the interaction between RBS and FOIS interference underscores the significance of laser pulse self‐correction in mitigating parasitic interference, offering a novel idea to address this issue. We proposed a PRN phase modulation method that aim to diminish parasitic interference by reducing self‐correction value of pulse, while preserving the monochromaticity. The new approach achieves a remarkable noise suppression of 21.3 dB with a leading fiber of 3.3 km.

Experimental findings indicate that the suppression effect increases with the order of the Gold pseudorandom code. This phenomenon can be rationalized by the fact that higher‐order PRN codes possess mathematical statistical characteristics closer to Gaussian white noise, resulting in sharper correlation peaks in their autocorrelation function. Conversely, the chip interval of the PRN has a marginal effect on the suppression effect. While PRN phase modulation can attenuate RBS by reducing parasitic interference intensity, complete elimination remains elusive, leading to residual background noise elevation. Consequently, endeavors to entirely mitigate the impact of RBS are ongoing.

## 3 Methods

### 3.1 Experimental Device Characterization

The laser employed in the experiments originates from RIO and boasts a wavelength of 1553.25 nm. Its intensity noise and phase noise capabilities stand at −120 $\mathrm{dB}\sqrt{\mathrm{Hz}}@$1 kHz and −110 $\mathrm{dB}\sqrt{\mathrm{Hz}}@$1 kHz respectively, laying a robust foundation for achieving minimal system background phase noise. The output pigtail is constructed from polarization‐maintaining fiber. The AOM is sourced from G&H and offers an extinction ratio exceeding 50 dB. The CIF features an all polarization‐maintaining fiber structure. Fiber optical circulators from FiberHome Co., Ltd, coupled with single‐mode pigtail fibers, are integrated into the sensor setup, with the coupling ratio set at 5%. The two FRMs, manufactured by Lightpromotech Co., Ltd, exhibit a reflectivity surpassing 99%. To fine‐tune the received laser intensity, a manually adjustable optical attenuator is employed. The DAQ facilitates the sampling of received signals and generation of modulation signals. For the PRN phase modulation experiment, an arbitrary waveform generator (AWG, SPECTRUM M4i. 6631) serves as the signal generator, while the EOM is a LiNbO_3_ phase modulator boasting a maximum modulation frequency of 10 GHz.

### 3.2 Demodulation Method of FOIS

**Figure 8:**
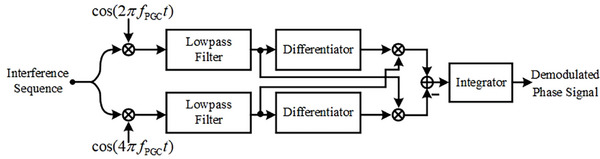
PGC‐DCM processing procedure.

The FOIS interference is demodulated utilizing the PGC method to nullify random phase shifts.^[^ ^55^, ^56^, ^57^ ^]^ As depicted in Figure 6, a cosine modulation signal is applied to the short arm of the CIF through the PZT ring, with a modulation amplitude of 2.37. The sampled interference is demodulated using the differential cross multiplexing (DCM) method, with the signal processing workflow outlined in **Figure** **8**.

### 3.3 Method to Generate the Pseudorandom Code

The pseudorandom binary sequence (PRBS) is employed to implement common‐mode phase modulation on the interrogation light pulses, effectively reducing their coherence and suppressing residual RS noise. PRBS exhibits self‐correlation and cross‐correlation statistical features akin to Gaussian white noise^[^ ^58^, ^59^ ^]^ and is characterized by a predetermined number of elements in its sequence. Therefore, PRBS can be systematically generated and replicated based on a specific algorithm, a practice commonly utilized in spread spectrum communication.

**Figure 9:**
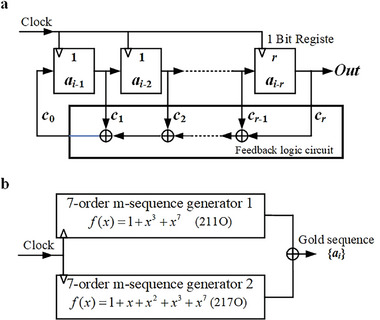
Principle of PRBS generation. a) Using the LFSR to generate the m‐sequence generation. b) Using the “modulo 2 sum” to generate the Gold code sequence.

The m‐sequence and Gold code sequence represent the most prevalent PRBSs, with the latter comprising the “modulo 2 sum” of two same‐order preferred pairs of the former. The principle of utilizing the linear feedback shift register (LFSR) to generate the m‐sequence is illustrated in **Figure** **9**a.

The *r* single‐bit registers depicted in Figure 9a store the continuously updated *r* sequence elements, with *c_i_* ∈ {0, 1} representing the feedback coefficient. When the initial values of the registers are not all zero, the distribution of elements in the generated sequence is solely determined by the feedback coefficient *c_i_*. A polynomial *f*(*x*)in the binary domain {0, 1}characterizes the LFSR network as follows:

(14) $$f\left(x\right)=1+c_{1}x+c_{2}x^{2}+\cdots +c_{r}x^{r}c_{i}\in \left\{0,1\right\}.$$

where *f*(*x*) is termed to as the characteristic polynomial of the *r*th‐order LFSR network. If the period of *f_m_* (*x*)generated sequence satisfies*N* = 2^*r*^ − 1, the output sequence is referred to as *r*th‐order maximum period linear shift register sequence, commonly referred to as an *r*th‐order m‐sequence.

The preferred pairs of 7th‐order m‐sequences with polynomials *f*(*x*) = 1 + *x* + *x* ^2^ + *x* ^3^ + *x* ^7^and*f*(*x*) = 1 + *x* ^3^ + *x* ^7^are adopted. The principle of generating the Gold code sequence using the “modulo 2 sum” method is illustrated in Figure 9b.

The Gold code sequence is utilized to implement phase modulation of the interrogation light pulses. The interrogation light pulse has a width of 490 ns, and the AWG can output a minimum chip interval of 0.8 ns, resulting in a maximum number of chips of 612. Consequently, the maximum Gold code sequence order is 9, corresponding to a total of 511 chips. In the experiments, the preferred pairs of 6th, 7th, and 9th order m‐sequences are employed to generate the Gold code sequences. The primitive polynomials, represented in octal form, are detailed in **Table** **2**.

**Table 2:** Primitive polynomials of the preferred pairs of m‐sequences for the 6th, 7th, and 9th order Gold code sequences used in the experiment.

Order of Gold code	Number of chips	Primitive polynomial of the preferred pair of m‐sequence
6	63	103O/147O
7	127	211O/217O
9	511	1021O/1131O

## 4 Conclusion

In this work, the parasitic interference in FOIS with common leading fiber have been systematically expatiated. The self‐correlation of pulse is the key parameter for quantitative calculation of intensity according to the theoretical model based on the wave theory. The PRN phase modulation which originated in the field of communication was employed to reduce parasitic interference through adjusting the correlation of pulse. A remarkable noise suppression of 21.3 dB with a leading fiber of 3.3 km proved the usefulness of the PRN method. This method represents a revolutionary milestone in the development of large‐scaled optical fiber sensing system. Better performances of FOIS are straightforwardly possible in the future with further exploration of PRN sequences.

## Conflict of Interest

The authors declare no conflict of interest.

## Author Contributions

Q.H. and F.S. contributed equally to this work. Q.H. and F.S. conceived the idea and performed the experiments together with Y.B., Y.S. and Yue Qi. L.M. developed the theory and performed the data analysis. The manuscript was written by L.M. and Y.Y. with assistance from all authors. X.Z. supervised this research project. J.S. provided useful suggestions on self‐correction self‐correlation function.

## Data Availability

The data that support the findings of this study are available from the corresponding author upon reasonable request.
